# Rice Yellow Stunt Nucleorhabdovirus Matrix Protein Mediates Viral Axonal Transport in the Central Nervous System of Its Insect Vector

**DOI:** 10.3389/fmicb.2019.00939

**Published:** 2019-05-09

**Authors:** Haitao Wang, Juan Wang, Qian Zhang, Tianbao Zeng, Yuemin Zheng, Hongyan Chen, Xiao-Feng Zhang, Taiyun Wei

**Affiliations:** ^1^Fujian Province Key Laboratory of Plant Virology, Fujian Agriculture and Forestry University, Fuzhou, China; ^2^State Key Laboratory for Ecological Pest Control of Fujian and Taiwan Crops and College of Life Science, Fujian Agriculture and Forestry University, Fuzhou, China

**Keywords:** RYSV, *Nucleorhabdovirus*, leafhopper, infection route, CNS, axonal transport

## Abstract

Persistently transmitted plant viruses encounter multiple membrane and tissue barriers in the process of completing their infection routes within their insect vectors. Some of these viruses have been reported to overcome the elaborate barriers of the central nervous system (CNS) to travel through the nervous tissues, but the specific mechanisms of this process remain unknown. Here, we report the axonal transport mechanism of rice yellow stunt virus (RYSV), a nucleorhabdovirus, in the CNS of the green rice leafhopper (*Nephotettix cincticeps*). The infection route of RYSV in the internal organs of its insect vector after ingestion of the virus was investigated by immunofluorescence microscopy. RYSV was first detected in the epithelial cells of midgut regions, from where it proceeded to the nervous system, and finally into the salivary glands. We then utilized immunofluorescence and electron microscopy to investigate the distribution of RYSV particles within the leafhopper CNS, demonstrating that non-enveloped viral particles distributed along the microtubule-based neurofilaments in the axon cytoplasm following the direct interaction of leafhopper α-tubulin with the RYSV M protein. Tubulin inhibitors inhibited the dissemination of RYSV to the CNS, then into the salivary glands in leafhoppers. We therefore describe a mechanism of plant virus transport through CNS axons as an alternative means of rapid viral dissemination in an insect vector.

## Introduction

Plant viruses cause significant agricultural losses in temperate, subtropical, and tropical regions. Almost 80% of plant viruses are persistently, semi-persistently, or non-persistently transmitted by aphids, leafhoppers, whiteflies, and other arthropod vectors (Hogenhout et al., 2008). In recent decades, the transmission route of persistently transmitted plant viruses in their insect vectors has been well documented. It is generally considered that, once ingested by their insect vectors, these viruses establish their primary infection in the alimentary canal epithelium, then pass directly through the basal lamina from the infected epithelial cells toward the visceral muscles or cross the intercellular junctional complexes for cell-to-cell spread (Hogenhout et al., 2008; Bragard et al., 2013; Blanc et al., 2014; Wei and Li, 2016; Whitfield et al., 2018). The viruses then spread to the hemolymph and eventually invade either the salivary glands to be horizontally transmitted to healthy plants, or the female ovary to be vertically transmitted to the offspring (Hogenhout et al., 2008; Bragard et al., 2013; Blanc et al., 2014; Wei and Li, 2016). Thus, the midgut, salivary gland, and ovary are the three main transmission barriers for persistently transmitted plant viruses in their insect vectors (Hogenhout et al., 2008; Wei and Li, 2016).

The mechanisms by which these viruses overcome the three transmission barriers have also been extensively investigated. The actin-based tubular structures formed by the non-structural proteins of rice reoviruses are believed to enable the virus to overcome the midgut or salivary gland barriers in their insect vectors (Chen et al., 2012; Jia et al., 2014; Wei and Li, 2016; Mao et al., 2017). In addition, some plant reoviruses, such as wound tumor virus (WTV), rice dwarf virus (RDV) and plant rhabdoviruses, including maize mosaic virus (MMV) and maize fine stripe virus (MFSV), are neurotropic and can systemically infect the nervous system of their insect vectors (Hirumi et al., 1967; Ammar and Hogenhout, 2008; Todd et al., 2010; Chen et al., 2011). Although plant viruses can infect, propagate in, and spread through the nervous system of vector insects, the molecular mechanisms involved in this transport within the neurons remain unknown.

The central nervous system (CNS) is a vital organ involved in regulating the vital physiological activities of insects (Osorio et al., 1997). The axons in charge of nerve impulse transmission are specialized protrusions containing more than 95% of the neuronal cytoplasm. Microtubules, actin filaments, and neurofilaments constitute cytoskeleton structures of neurons. The axon microtubules provide specific routes for transporting cytoplasmic cargos driven by dynein or kinesin motor protein through the nervous system (Mitchison and Kirschner, 1984; Osorio et al., 1997; Kapitein and Hoogenraad, 2011). As early as 50 years ago, electron microscopic observations showed that the plant reovirus WTV was distributed within the axons of the CNS in its leafhopper vector (Hirumi et al., 1967). Subsequently, immunofluorescence microscopic observations showed that two plant rhabdoviruses, MMV and MFSV, could spread from the initially infected midgut region to the CNS and eventually invade the salivary glands, which are innervated by salivary nerves of leafhoppers projecting from the suboesophageal ganglion (SEG) or the stomatogastric nervous system (SNS) (Sogawa, 1971; Penzlin, 1985; Ali, 1997; Hartenstein, 1997; Ammar and Hogenhout, 2008; Todd et al., 2010). It therefore seems that neurotropic plant viruses may undergo long-distance transport in the insect CNS, a process that is believed to facilitate viral transmission. Whether rapid axonal transport is involved in the neurotropic transmission of plant rhabdoviruses through insect vectors is still unknown.

Rice yellow stunt virus (RYSV) is the only nucleorhabdovirus (family Rhabdoviridae) known to infect rice. It was first described in 1965 in China and causes economically serious reductions in rice yields in Asia (Chiu et al., 1965; Fan et al., 1965). The RYSV genome contains seven open reading frames (ORFs) in the order 3′-N-P-3-M-G-6-L-5′, which encodes seven proteins: nucleoprotein (N), phosphoprotein (P), putative movement protein P3 (3), matrix protein (M), glycoprotein (G), RNA silencing suppressor P6 (6) and large RNA polymerase (L) (Fang et al., 1994; Luo and Fang, 1998; Luo et al., 1998; Huang et al., 2003, 2005). The N proteins of different rhabdoviruses have a highly conserved function and encapsidate the entire RNA genome and tightly package it into an RNase-resistant core (Luo et al., 2007). The P proteins play key roles in virus replication by interacting with the N and L proteins (Jackson et al., 2005, 2008; Wang et al., 2018). The M proteins of rhabdoviruses are associated with the condensation of the ribonucleoprotein complex cores and the formation of bullet-shaped non-enveloped virion particles (Jayakar et al., 2004; Kuzmin et al., 2009), while the M protein of the most extensively studied nucleorhabdovirus, sonchus yellow net rhabdovirus (SYNV), appears to be required for the long-distance movement of the virus within the plant (Wang et al., 2015). The G proteins of rhabdoviruses, which are exposed on the surface of enveloped virion particles, are responsible for virus entry into host cells through specific receptor binding (Gaedigk et al., 1986; Jayakar et al., 2004; Jackson et al., 2005). How these viral proteins are involved in the neurotropic spread of plant rhabdoviruses in insect vectors is still unknown.

RYSV is transmitted by leafhoppers in a persistent manner (RYSV can replicate and induce viral inclusions in the cells of its insect vector), however, the infection route of this virus within its insect vector remains unclear. Here, we used RYSV and its main vector, the leafhopper *Nephotettix cincticeps* (Hemiptera, Delphacidae), to explore how a plant rhabdovirus utilizes axonal transport within its insect vector. We report that the interaction of RYSV M protein with the insect axonal microtubules mediates the rapid transmission of the bullet-shaped non-enveloped virions along the long axonal microtubule filaments in the insect vector CNS, facilitating persistent virus transmission.

## Materials and Methods

### Cell, Insects, Virus, and Reagents

Continuous vector cell monolayer (VCM) cultures were developed from embryonic fragments of *N. cincticeps* leafhoppers and maintained on a growth medium at 25°C, as described previously (Kimura, 1984). The leafhoppers were collected from Yunnan Province in southern China. The insects were screened, and a non-viruliferous colony was reared on rice seedlings in clear containers in a controlled environment at 28°C. The viral copies (2.144E+06) of RYSV solution were calculated as the log of the copy number per microgram of purified virus RNA by mapping the Cq value to the standard curve of RYSV *N* gene clone vector (*y* = −3.4436+41.546, *R*^2^ = 0.994). Purified virus was stocked in different tubes at −80°C in a refrigerator for later use.

Rabbit polyclonal antisera against the RYSV M and P proteins were prepared as described previously (Wang et al., 2018). IgGs were purified by Protein A IgG Binding Buffer (Thermo, product number, 21001) and IgG Elution Buffer (Thermo, product number, 21004) from specific polyclonal antibodies following the manual instructions and conjugated directly to fluorescein isothiocyanate (FITC) or to rhodamine (Thermo Fisher Scientific), according to the manufacturer’s instructions. Colchicine (MCE, HY-16569) and vincristine sulfate (MCE, HY-N0488) were used as microtubule inhibitors or disrupting agents. The α-tubulin antibody (Sigma-Aldrich, T6199), a general marker of neural structures, was used to label the nerves of *N. cincticeps*.

### Transmission Electron Microscopy

The CNSs of RYSV-infected adult instar *N. cincticeps* were dissected, fixed with 2.5% glutaraldehyde (Sigma, G5882) in 0.01 M phosphate-buffered saline buffer (PBS, pH, 7.2) at 4°C overnight and then post-fixed with 1% osmium tetroxide for 1.5 h at room temperature, dehydrated with a series of different concentrations of ethanol, and then embedded with Spurr low viscosity embedding resin at 70°C for 24 h. Ultrathin sections of the CNSs and VCMs were prepared with an ultramicrotome (Leica UC7) and double stained with 2% uranyl acetate and 3% lead citrate. In total, 126 ultrathin sections from 42 viruliferous CNS of *N. cincticeps* individuals (3 sections for each leafhopper) were observed. For the immunoelectron microscopy, the CNSs of RYSV-infected adult instar *N. cincticeps* were dissected, fixed with 2% glutaraldehyde (Sigma, G5882) and 2% paraformaldehyde (PFA, Sigma, 158127) in 0.01 M PBS (pH, 7.2) at 4°C overnight, dehydrated with a series of different concentrations of ethanol at −20°C, and then embedded with LR GOLD resin (Agar Scientific, AGR1284) at −20°C for 96 h under ultraviolet light. The ultrathin sections of leafhopper CNS were incubated with protein-M-specific IgG from rabbit and immunogold-labeled using goat antibodies against rabbit IgG conjugated with 15 nm gold particles (Sigma-Aldrich), as described previously (Mao et al., 2017). The samples were then observed under an electron microscope (Hitachi H-7650).

### Immunofluorescence Microscopy

Immunofluorescence microscopy was used to elucidate the distribution of viral antigens in the bodies of leafhoppers that had ingested RYSV from diseased plants as a means to study the infection route of RYSV. Second-instar *N. cincticeps* nymphs were fed RYSV-infected rice plants for 2 days and then transferred to healthy rice seedlings. At various time points (2, 4, 6, 8, and 10 days) after their exposure to the virus, the digestive tracts and CNSs of 30 *N. cincticeps* individuals were dissected at each time point, fixed in 4% PFA in 0.01 M PBS at room temperature for at least 8 h, and permeabilized at room temperature in 4% Triton X-100 in 0.01 M PBS buffer for 24 h. The internal organs were then immunolabeled with viral-antigen-specific IgG conjugated to rhodamine (virus-rhodamine, V-R, the conjugated antibody was diluted with albumin from bovine serum, with the final concentration approximately to 0.1 mg/ml) and Alexa Fluor^TM^ 488 Phalloidin (Thermo Fisher Scientific, A12379, 1:200). As controls, the internal organs of that *N. cincticeps* fed on healthy rice plants were dissected and treated in the same way. The samples were then examined using a Leica TCS SP5II confocal microscope (in order to avoid fluorescence interference caused by close wavelength, two independent channels were used to observe the fluorescence signals).

The *N. cincticeps* VCMs that reached 80% confluence were washed with the His-Mg solution (0.1 M histidine and 0.01 M MgCl_2_, pH 6.2), then inoculated with RYSV solution at an MOI of 0.4 for 2 h. The cells were then washed with His-Mg and covered with growth medium before being fixed and immunolabeled with α-tubulin-FITC antibody (α-tubulin-F, Sigma, F2168, 1:50) and protein-M-specific IgG conjugated to rhodamine (M-rhodamine, M-R, the conjugated antibody diluted with albumin from bovine serum, with a final concentration of approximately 0.1 mg/ml). The samples were then examined with a confocal microscope.

### Baculovirus Expression of the RYSV M Protein

A recombinant baculovirus expression system was used to study the localization of the M protein expressed in Sf9 cells, as described previously (Jia et al., 2012). A baculovirus vector expressing a recombinant M protein fused with a 6 × His tag (M-His) was transformed into *Escherichia coli* DH10 Bac cells (Thermo Fisher Scientific) to prepare the recombinant bacmid. The recombinant bacmids DNA were transfected into Sf9 cells using Cellfectin^TM^ II Reagent (Thermo Fisher Scientific, 10362100), according to the manufacturer’s instructions. We collected the culture supernatant 3 days after the recombinant bacmids transfected. After 30 h of incubation with the M recombinant baculovirus (culture supernant), the infected Sf9 cells were fixed in 4% PFA, immunolabeled with an 6 × His-tag^®^ antibody (FITC) (Abcam, ab1206, 1:200) and α-tubulin antibody (Sigma-Aldrich, T6199, 1:200), then analyzed using an immunofluorescence microscope.

### Yeast Two-Hybrid Assay

A yeast two-hybrid assay was performed using the Matchmaker Gal4 Two-Hybrid System 3 (Takara Bio), according to the manufacturer’s instructions. The RYSV *M* gene was amplified and cloned into the bait vector pGBKT7, and the *α-tubulin* gene from *N. cincticeps* (Genbank number is KU196671.1) was cloned into the prey vector pGADT7. The recombinant vectors pGBKT7-M and pGADT7-α-tubulin; the positive control pGBKT7-53/pGADT7-T; the negative controls pGBKT7-Lam/pGADT7-T, pGBKT7-M/pGADT7, and pGBKT7/pGADT7-α-tubulin; and the vectors supporting the interaction of the G protein with α-tubulin (pGBKT7-G/pGADT7-α-tubulin) and the N protein with α-tubulin (pGBKT7-N/pGADT7-α tubulin) were each co-transformed into the AH109 yeast strain. The β-galactosidase activity (indicative of successful cloning) was assessed on SD/-Leu/-Trp/-His/-Ade/X-a-gal agar culture medium plates.

### GST Pull-Down Assay

A GST pull-down assay was performed to detect the interaction of the RYSV M protein and *N. cincticeps* α-tubulin. The RYSV *M* gene was amplified and cloned into the vector pGEX-3X, which includes a GST tag (M-GST). The *α-tubulin* gene of *N. cincticeps* was cloned and inserted into a His-fused vector, pDEST17 (His-α-tubulin). The constructed plasmids pDEST17-α-tubulin, pGEX-3X-M, and pGEX-3X (GST) were separately expressed in the *E. coli* strain BL21. The lysates of cells containing the pGEX-3X-M (M-GST) and pGEX-3X (GST) vectors expressed proteins and were incubated with Glutathione Sepharose beads (Amersham) for 4 h at 4°C. The beads were rinsed with 0.01 M PBS to remove the redundant proteins, then they were incubated with the lysates of cells containing pDEST17 (His-α-tubulin) protein for a further 4 h at 4°C. The mixtures were then washed with elution buffer and visualized in an immunoblotting assay using GST-tag and His-tag antibodies (Abcam), respectively.

### Effects of Tubulin Inhibitors on Viral Infection and Accumulation in RYSV-Infected *N. cincticeps*

To examine the influence of the tubulin on the RYSV infection rate, tubulin-inhibitor or disrupting reagents were serially diluted with 0.01 M PBS (pH 7.2) and applied to the VCMs at 2 h post-RYSV inoculation (at a MOI of 0.4) for 48 h. The cells were then washed with PBS to remove the inhibitors. At 48 hpi, the VCMs were fixed in 4% PFA, permeabilized with 0.2% Triton X-100, then incubated with anti-α-tubulin antibody (Sigma-Aldrich, T6199, 1:200) and M-R at room temperature for 1 h. The samples were observed using a confocal microscope to quantify the RYSV infection levels, and the mean values were calculated based on three replicates. The accumulation of viral proteins was analyzed using immunoblotting assays with RYSV N-specific or RYSV M-specific IgGs.

To further analyze the role of tubulin in the dissemination of RYSV in *N. cincticeps*, third-instar nymphs were microinjected with either 200 nL colchicine (50 μg/ml) diluted with RYSV inoculum, or with a RYSV solution diluted with the same volume of 0.01 M PBS (pH 7.2) as a control. The 500 microinjected insects were reared on healthy rice seedlings for each treatment each time. To trace the infection of RYSV in the CNS, a group of 30 leafhoppers from each treatment were dissected at different days (3, 6, and 8 days) post-microinjection and fixed, permeabilized, and immunolabeled with α-tubulin antibody (Sigma-Aldrich, T6199, 1:200) or virus-antibody-rhodamine (V-R). To further measure the effect of the tubulin inhibitor colchicine on the transcript levels of the RYSV *M* (Forward primer: CCCGATCATGAAGCCACTAC, Reverse primer: CTTATTGTAGCACCCACCCC. Efficiency: 85.785%) and *N* genes (Forward primer: AGTATGCCCAACTTGCCAGG, Reverse primer: CATTCGTTCAACCGGCATCC. Efficiency: 95.165%), RNAs from the heads and ganglion components of the two treatment groups of insects were extracted at 1, 3, 5, 7, 9, and 11 days post-microinjection and analyzed using an RT-qPCR assay. The qPCR program was divided into three stages (hold stage with two steps, 50°C for 2 min and 95°C for 10 min; PCR stage with 40 PCR cycles; and melt curve stage with three stages, 95°C for 15 s, 60°C for 1 min and 95°C for 1 s) and conducted by a QuantStudioTM 5 Real-Time PCR machine. The detected transcript levels were normalized with respect to an internal control gene, *actin* (Forward primer: GGGATACAGTTTCACCACG, Reverse primer: GACACCTGAATCGCTCGT. Efficiency: 93.164%) and were estimated using the 2^−ΔΔCt^ (cycle threshold) method. The gene transcript levels were set to 1.0 at 1 days post-microinjection and analyzed different data, respectively. The results were analyzed from three biological repeats. Additionally, the total proteins from the heads of insects exposed to the two treatments were extracted, and the accumulation of RYSV was analyzed in an immunoblotting assay with N-specific or P-specific antibodies at 8 days post-microinjection.

### Statistical Analysis

All data were analyzed for statistical differences using SPSS 19.0. Multiple comparisons of the means were conducted using a one-way analysis of variance and Tukey’s honest significant difference (HSD) test.

## Results

### RYSV Infection Route Within the Body of *N. cincticeps*

**FIGURE 1:**
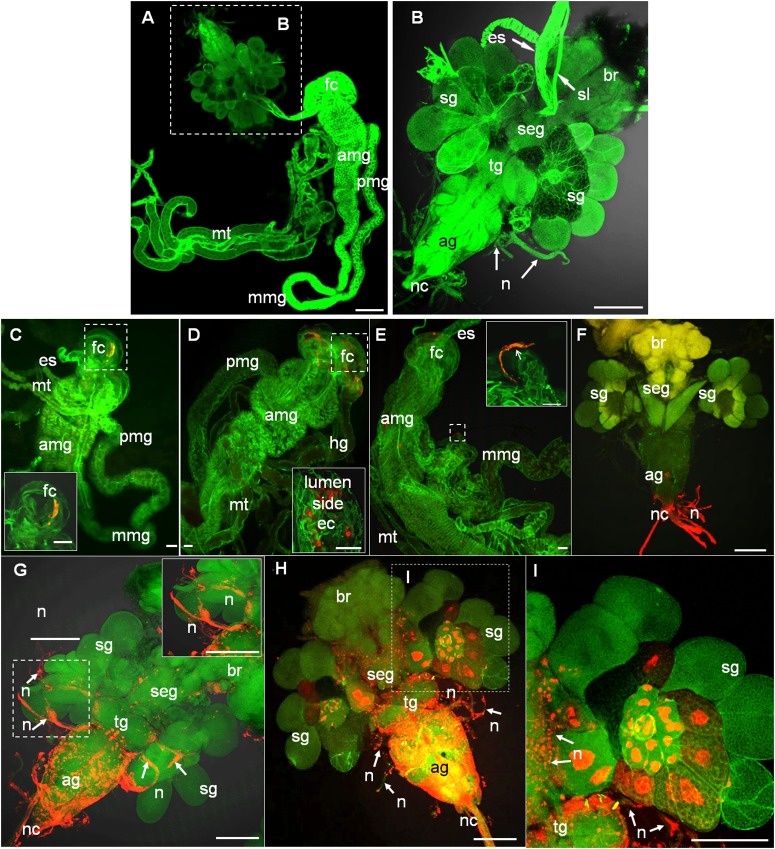
Infection route of RYSV within the internal organs of its leafhopper vector *N. cincticeps*. **(A,B)** Anatomical and morphological features of the digestive system and CNS of adult instar *N. cincticeps*, the head region was magnified in **(B)**. **(C–I)** Infection route of RYSV through the internal organs of *N. cincticeps*. Second-instar *N. cincticeps* nymphs were fed on RYSV-infected rice plants for 2 days and then transferred to healthy rice seedlings. All organs or tissues were immunolabeled for RYSV using Alexa Fluor (red) and stained for actin with phalloidin (green), and then examined using confocal microscopy. **(C)** At 2 days padp, RYSV was detected in the filter chamber. Inset, higher magnification of the image in the boxed area. **(D)** At 4 days padp, the virus spread from the filter chamber to the anterior part of midgut. **(E)** A nerve-like structure surrounding the mid-part of the midgut was infected by RYSV at 4 days padp. **(F)** At 6 days padp, RYSV was detected in the nerve cord of the CNS. **(G)** At 8 days padp, RYSV infected nerves of salivary glands projecting from the CNS. **(H,I)** By 10 days padp, RYSV was detected in the CNS and the salivary gland. ag, abdominal ganglion; amg, anterior part of midgut; br, brain; ec, epithelial cells; es, esophagus; fc, filter chamber; hg, hindgut; mmg, mid-part of the midgut; mt, Malpighian tubules; n, nerve; nc, nerve cord; pmg, posterior part of midgut; rt, rectum; seg, subesophageal ganglion; sg, salivary gland; sl, suspensory ligament; tg, thoracic ganglion. Scale bars, 100 μm.

To trace the infection route of RYSV within infected leafhoppers, we dissected the internal organs of 30 leafhoppers collected at five time points (2, 4, 6, 8, 10 post-first access to the diseased plants, or padp) and examined them using immunofluorescence microscopy, respectively. In leafhoppers, the alimentary canals consist of the esophagus, a filter chamber, the midgut, and the hindgut (Figure 1A–E); the CNS comprises the brain, subesophageal ganglion, thoracic ganglion, and abdominal ganglion (Figure 1A); and the salivary glands consist of two paired sets of principal and accessory glands, located on either side of the thoracic ganglion (Figure 1B; Sogawa, 1971).

We found that as early as 2 days padp, RYSV had accumulated in a specific region of the filter chamber in 73% of leafhoppers tested (Figure 1C and Table 1), suggesting that RYSV ingested by leafhoppers from diseased rice plants crossed the microvilli into the epithelial regions of the filter chamber. At 4 days padp, the viral antigens had spread from the filter chamber to the anterior part of the midguts in about 17% of leafhoppers (Figure 1D and Table 1), and RYSV occasionally presents in the nerve-like structures surrounding the midgut (Figure 1E). At 6 days padp, RYSV had spread extensively in the filter chamber and the anterior part of the midgut in 46% of leafhoppers. For the first time, RYSV was detected in neural tissues, including the nerve cords of the abdominal ganglions in 48% of viruliferous leafhoppers (Figure 1F and Table 1). Despite this, RYSV was not detectable in the salivary glands adjacent to the abdominal ganglion (Figure 1F and Table 1). At 8 days padp, RYSV had spread from the infected CNS to the adjacent principal salivary glands through the nerve fibers (Figure 1G and Table 1), while we detected the immunofluorescence signals of RYSV in the hemolymph (Supplementary Figure S1). At 10 days padp, viral antigens were observed in the midgut, abdominal ganglions, and the other parts of the CNS of most viruliferous leafhoppers tested (Figure 1H,I and Table 1).

**Table 1:** Occurrence of viral antigens in various organs and tissues of *N. cincticeps* at different times following their first access to diseased plants (padp).

Days padp	No. of insects positive for virus antigens in the specified tissue (*n* = 30/day)							
	Fc-ec	Fc-vm	Amg	Mmg	Pmg	Hg	Sg	Ag
2	22	0	1	0	0	0	0	0
4	23	0	4	0	0	0	0	0
6	21	0	14	1	0	0	0	10
8	22	4	14	1	1	1	2	14
10	21	4	20	1	4	4	7	17

*Second-instar nymphs of N. cincticeps were fed RYSV-infected rice plants for 2 days then transferred to healthy rice seedlings. After different periods of time following their exposure to the diseased plants, the internal organs of 30 individuals were immunolabeled for viral antigens and counted. Fc-ec, filter chamber epithelial cells; Fc-vm, filter chamber visceral muscle; Amg, anterior part of midgut; Mmg, mid part of midgut; Pmg, posterior part of the midgut; Hg, hindgut; Sg, salivary gland; Ag, abdominal ganglion; padp, post-first access to diseased plants.*

### Distribution of RYSV in the *N. cincticeps* CNS

**FIGURE 2:**
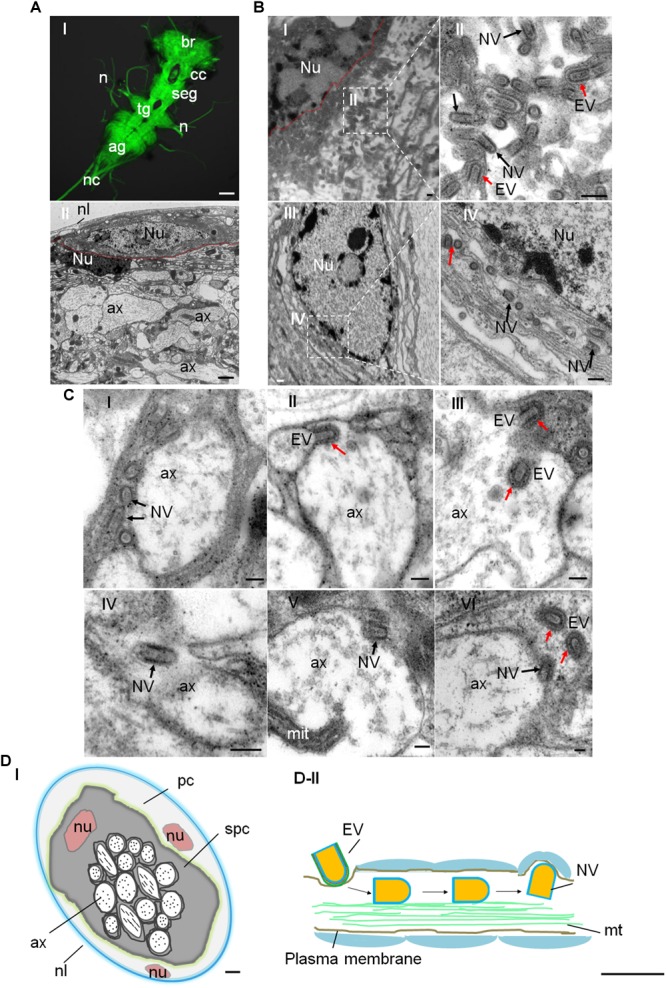
Distribution of RYSV within the CNS of *N. cincticeps*. **(A-I)** A dissected adult instar *N. cincticeps* CNS, immunolabeled with α-tubulin antibody (green) and examined using a confocal microscope. The *N. cincticeps* CNS consists of the brain (in the head), circum-esophagus connectives, subesophageal ganglion, thoracic ganglion, abdominal ganglion, and the nerve cord. Scale bars, 100 μm. **(A-II)** Transmission electron micrograph showing the transverse abdominal ganglion of adult instar *N. cincticeps*. Scale bar, 2 μm. **(B-I)** Virions matured and budded from the nucleus of the perineurium glial cell of abdominal ganglion. Scale bar, 200 nm. **(B-II)** Magnified image of the boxed area in **(B-I)**. Scale bar, 100 nm. **(B-III)** RYSV particles budded from the nucleus and dispersed along the fiber networks in the neuropil of the thoracic ganglion. **(B-IV)** Magnified image of the boxed area in B-III. **(C)** The process of RYSV viral particles invading the axons of the CNS of *N. cincticeps*. The early infection stage is illustrated, depicting RYSV particles attached to the axon in **(C-I)** and RYSV particles enveloped with a membrane invading into axon in **(C-II)**. The trafficking of RYSV viral particles through the center of the CNS axons **(C-III,IV)**, and the RYSV particles attached to the axon membrane and released from the axons into the subperineurium glial cell in **(C-V)** and **(C-VI)**. Scale bars, 100 nm. **(D-I)** Model of the transverse CNS in *N. cincticeps*. The entire ganglion is enclosed by the neural lamella and a perineurium glial cell. The subperineurium cells are located at the periphery, while the axons occupy the central core of the ganglion. Scale bar, 10 μm. **(D-II)** Model of RYSV viral particle trafficking in an axon of the *N. cincticeps* CNS. RYSV particles attach to the membrane and invade the axons. The interaction of the structural protein M with the tubulin in the axons results in the rapid delivery of the viral particles throughout the CNS. Scale bars, 200 nm. ag, abdominal ganglion; ax, axon; br, brain; cc, circum-esophagus connectives; cor, cortex; mit, mitochondria; mt, microtubule; n, nerve(s); nc, nerve cord; nl, neural lamella; nu, nucleus; pc, perineurium glial cells; seg, subesophageal ganglion; spc, subperineurial glial cells; tg, thoracic ganglion; Red arrows, EV, enveloped viral particles; Black arrows, NV, non-enveloped viral particles.

We used electron microscopy to observe the distribution of RYSV in the CNS of viral infected *N. cincticeps.* The CNS ganglion is enclosed within the neural lamella and perineurium (Figure 2A-I,II). The ganglion cell bodies are situated at the periphery of each ganglion, while the neuropil occupies the central core (Figure 2A-II). The neuropil consists of a large network of fibers, usually surrounded by the cytoplasm of the inner glial cells, and the nerve axons extend out from the neuropil. The axoplasm (axon cytoplasm) contains a few mitochondria and numerous neurotubules composed of microtubules (Figure 2A-II). Electron microscopy showed that both enveloped and non-enveloped RYSV virions budded from the nuclei of the CNS ganglion cells, which are the site of viral replication and progeny virion assembly (Figure 2B-I,II). We observed that many non-enveloped virions were distributed along the fiber networks in the neuropil (Figure 2B-III,IV). The non-enveloped virions were frequently found in the axoplasm (Figure 2C), while it appeared that some enveloped virions could pass through the axon plasma membrane to enter the cell (Figure 2C-II,III). Figure 2C-II shows a single enveloped virion attached to the axonal membrane to form an invagination, from which it could enter the axoplasm. Furthermore, some non-enveloped virions could attach to the inner axonal membrane and exit the axon (Figure 2C-V,VI). To distinguish the virus with and without envelopment, we performed the immunoelectron microscopy assay with RYSV G-specific antibody (Supplementary Figure S3). We calculated that about 60% of virions in the axoplasm were non-enveloped (Supplementary Figure S2A-I). Besides, more non-enveloped viral particles were found in the exit site of CNS axons (Supplementary Figures S2A-II,III). These results indicated that RYSV used enveloped virions to enter the axon but may use non-enveloped virions to perform long-distance axonal transport in the axoplasm of CNS within its insect vector (Figure 2D-I,II).

### The RYSV M Protein Interacts With *N. cincticeps* Microtubules

**FIGURE 3:**
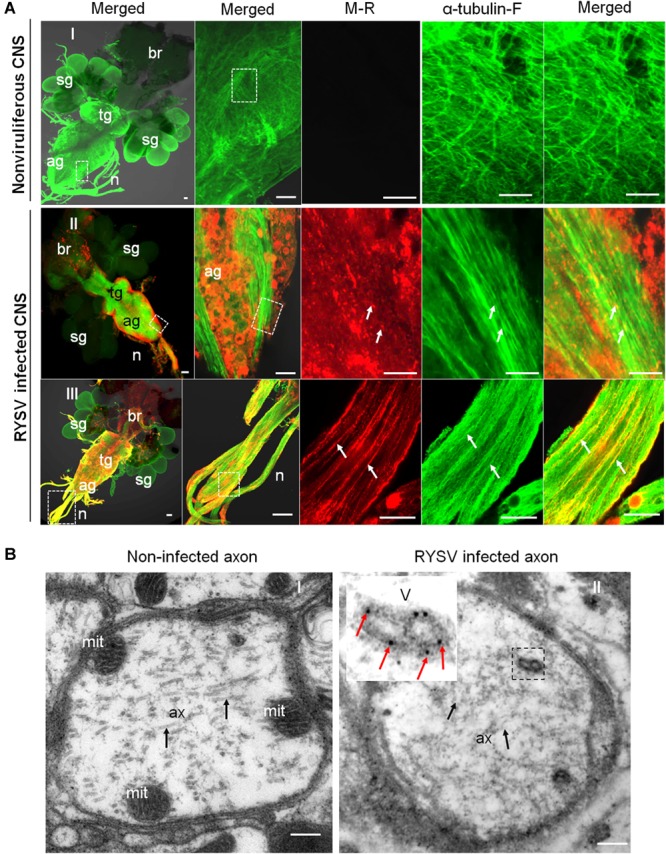
Localization of M protein in the CNS of RYSV infected *N. cincticeps*. **(A)** The localization of the RYSV M protein in the abdominal ganglion of viruliferous adult instar *N. cincticeps*. **(A-I)** CNS of non-infected *N. cincticeps* (control). **(A-II,III)** The RYSV M protein co-localized with α-tubulin in the abdominal ganglion **(A-II)** and the nerves **(A-III)** of RYSV infected *N. cincticeps*. ag, abdominal ganglion; br, brain; n, nerves; sg, salivary gland; tg, thoracic ganglion. White arrows indicate the co-localization of α-tubulin and RYSV M protein. Scale bars, 200 μm. **(B)** The viruliferous CNS of adult instar *N. cincticeps* was analyzed using immunoelectron microscopy. **(B-I)** Non-RYSV-infected samples (control). **(B-II)** The viruliferous CNS of adult *N. cincticeps* was immunolabeled with RYSV M specific antibodies, and indicated with gold particles. ax, axon; mit, mitochondria; V, virion(s). Black arrows, microtubule; Red arrows indicate gold particles. Scale bars, 100 nm.

We then investigated how the non-enveloped RYSV virions utilize axonal transport in the CNS of *N. cincticeps*. It is clear that the M proteins of rhabdoviruses play an important role in the assembly of mature virions, including the condensation of the ribonucleoprotein cores to form bullet-shaped non-enveloped virions (Jayakar et al., 2004; Kuzmin et al., 2009). We used immunofluorescence microscopy following immunolabeling with α-tubulin-specific antibodies conjugated to FITC (α-tubulin-FITC, α-tubulin-F) and M-specific antibodies conjugated to rhodamine (M-rhodamine, M-R) to reveal the co-localization of the RYSV M protein with the axon-associated microtubules in the different parts of the CNS (ag and nerves) of viruliferous *N. cincticeps* (Figure 3A-II,III). Our immunoelectron microscopy observations further confirmed that the M-specific antibodies recognized the outer capsid of the non-enveloped RYSV virions in the axon of the abdominal ganglion in viruliferous *N. cincticeps* (Figure 3B). The axonal transport of RYSV may therefore depend on a specific interaction between the RYSV M protein and the microtubules in insect nerve cells.

**FIGURE 4:**
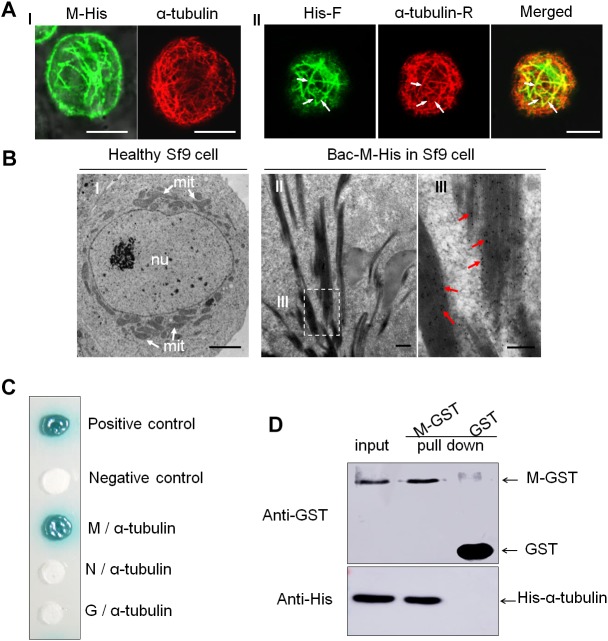
Interaction of RYSV M protein with α-tubulin of *N. cincticeps.* **(A)** Expressed RYSV M protein co-localized with α-tubulin in Sf9 cells. **(A-I)** The RYSV M protein formed tubular structures in Sf9 cells. (left) Samples were immunostained with M-specific antibodies at 30 h post-inoculation (hpi). (right) Healthy, uninfected Sf9 cells treated the same way served as the control, labeled with monoclonal α-tubulin antibodies conjugated with rhodamine (red, α-tubulin-R). **(A-II)** Samples were immunolabeled with rabbit polyclonal His antibodies conjugated with FITC (green, His-F) and α-tubulin-R. White arrows indicate the co-localization of α-tubulin and RYSV M protein. Scale bars, 15 μm. **(B)** Electron micrographs showing the subcellular localization of fusion protein M-His at 72 hpi in baculovirus-infected Sf9 cells. **(B-I)** Healthy, uninfected Sf9 cell (control). Scale bar, 2 μm. **(B-II)** Sf9 cells infected with baculovirus expressing M-His protein and immunolabeled with an M-specific rabbit polyclonal antibody as the primary antibody, then treated with goat-anti-rabbit IgG conjugated with 15 nm diameter gold particles. **(B-III)** Magnification of the boxed area in **(B-II)**. mit, mitochondria; nu, nucleus. Red arrows, gold particles. Scale bars, 100 nm. **(C)** Yeast two-hybrid assay analysis of the interaction between the RYSV M protein and *N. cincticeps* α-tubulin. β-galactosidase activity was detected on a SD/-Leu/-Trp/-His/-Ade/X-α-gal culture medium. **(D)** GST pull-down assay to detect the interaction of the RYSV M protein with *N. cincticeps* α-tubulin.

To determine whether RYSV M protein has an inherent ability to interact with these microtubules, we used a baculovirus system to express M protein in *Spodoptera frugiperda* cells (Sf9). Immunofluorescence microscopy showed that the M protein was distributed in filament-like structures in the cytoplasm and could co-localize with microtubules in the cytoplasm of Sf9 cells (Figure 4A). Our immunoelectron microscopy observations confirmed that the M-specific antibodies reacted specifically with the filament-like structures in the Sf9 cells (Figure 4B); thus, even in the absence of viral proliferation, the RYSV M protein was also associated with the microtubules.

A yeast two-hybrid assay further demonstrated that the RYSV M protein, but not the N or G proteins, specifically interacted with α-tubulin, the main component of the microtubules in the axoplasm (Figure 4C). A glutathione S-transferase (GST) pull-down assay confirmed that GST-fused RYSV M protein specifically bound to His-fused α-tubulin in *N. cincticeps*, whereas GST by itself did not (Figure 4D). These results demonstrated that the association of non-enveloped virions with microtubules in the insect axons was mediated by the specific interaction of the RYSV M protein and α-tubulin in *N. cincticeps*.

### RYSV Spreads Along the Microtubules of the Insect Vector Cells

**FIGURE 5:**
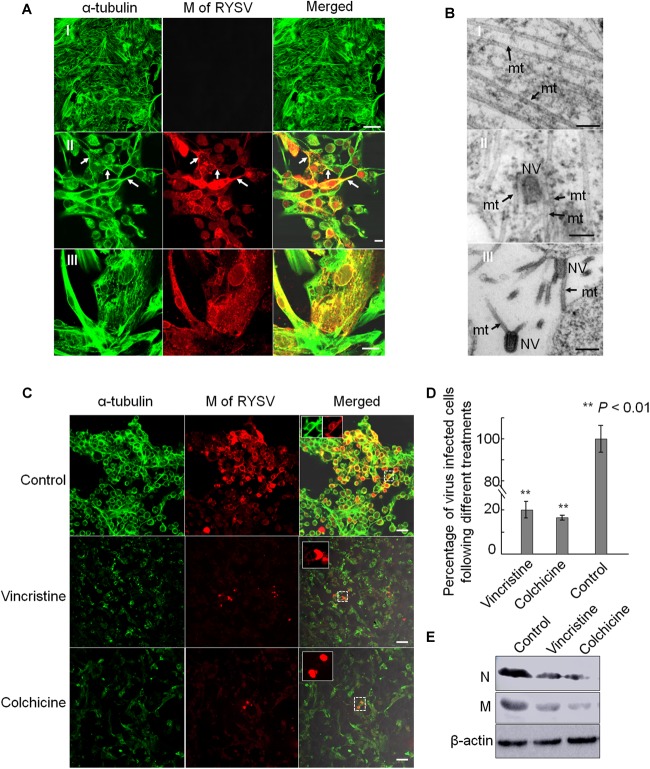
Association of RYSV with tubulin in *N. cincticeps* vector cell monolayers. **(A)** Immunofluorescence micrographs of the subcellular co-localization of the M protein with α-tubulin in vector cell monolayers (VCMs) at 72 h after viral inoculation in the non-infected VCM controls **(A-I)** and infected VCM cells **(A-II,III)**. White arrows indicate the co-localization of M with α-tubulin. Scale bars, 10 μm. **(B)** Electron micrographs showing RYSV viral particles closely associated with the microtubules in the *N. cincticeps* VCMs **(B-II,III)**, or the non-infected VCMs (control, **B-I**). Black arrows indicate microtubules. mt, microtubules; NV, Non-enveloped viral particle(s). Scale bars, 100 nm. **(C)** Tubulin inhibitors or disrupting reagents inhibited the infection of RYSV in infected VCMs. Scale bars, 10 μm. **(D)** Infection rates of RYSV following treatments with the tubulin inhibitors. Cells were imaged with an N-R antibody using a confocal microscope at 48 h after treatment. A total of 2500 cells per treatment were counted from three independent biological repeats. The data represent the means ± standard error (SE). *P* values were estimated using Tukey’s honest significant difference (HSD) test and Statistical significance is indicated by asterisks. **(E)** The abundance of viral proteins following treatment with the tubulin inhibitor was determined using an immunoblotting assay. N-specific antibodies and M-specific antibodies were used to detect N and M proteins in the VCMs treated with the various reagents at 96 hpi. The abundance of *N. cincticeps* actin, detected with the β-actin antibody, was used as the control.

We next addressed whether microtubules facilitate the long-distance spread of non-enveloped RYSV particles *in vitro*. We inoculated cultured VCMs derived from *N. cincticeps* with RYSV at a multiplicity of infection (MOI) of 0.4. At 72 h post-inoculation (hpi), we immuno-labeled the samples and found that RYSV had spread among the cultured cells to form infectious foci. The RYSV M protein co-localized with microtubules in the cytoplasm as well as with the cellular membrane and protrusions (Figure 5A). Examination of ultrathin sections of RYSV-infected VCMs at 72 hpi using electron microscopy revealed that some non-enveloped virions were closely associated with microtubules, approximately 25 nm in diameter, within the cytoplasm or in cellular protrusions (Figure 5B). This suggests that the microtubules may facilitate the spread of RYSV virions in insect vector cells.

We then used tubulin inhibitors to explore the influence of the microtubules on the spread of RYSV virions through the VCMs. In preliminary experiments, we tested a series of reagents with different concentrations to determine the optional concentration for treating the VCMs and decided to use vincristine (sulfate) and colchicine at concentrations of 10 μM and 5 μg/ml, respectively. These concentrations are optimized to avoid affecting the viability of the cells. We inoculated the VCMs with RYSV at an MOI of 0.4, added the inhibitors 2 h later, and continued to incubate the cells for a further 46 h. Using immunofluorescence microscopy, we showed that treatment with both the tubulin inhibitors resulted in the significant disassembly of the microtubules and a strong inhibition of viral infection (Figure 5C,D). Our immunoblotting assay results confirmed that the accumulation of RYSV N and M proteins decreased after the treatment (Figure 5E). Together, these results indicate that normal microtubule function is essential for RYSV trafficking in insect vector cells.

### RYSV Utilizes Axonal Transport in the CNS of Intact Insects

**FIGURE 6:**
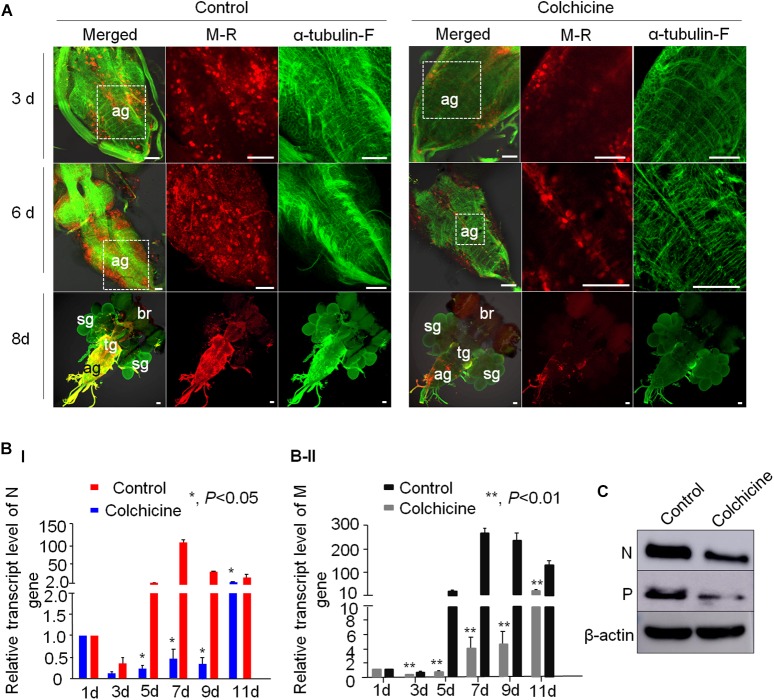
Tubulin inhibitor suppressed RYSV infection in the CNS of viruliferous *N. cincticeps*. **(A)** The effect of the tubulin inhibitor colchicine on live *N. cincticeps* was determined. The CNSs from the two groups were dissected at 3, 6, and 8 days after the microinjection and immunolabeled with α-tubulin antibody (α-tubulin-F; green) and virus-rhodamine (V-R; red). Scale bars, 200 μm. **(B)** The relative transcript levels of the *N* and *M* genes in the different samples outlined in **(A)** were analyzed using RT-qPCR at 1, 3, 5, 7, 9, and 11 days post-microinjection. *P*-values were estimated using Tukey’s honest significant difference (HSD) test and data represent means ± SE from three biological repeats and statistical significance is indicated by asterisks, *, 0.01 < *p* < 0.01. **(C)** The accumulation of RYSV N and P proteins in the CNS of *N. cincticeps* were detected using an immunoblotting assay with N-specific and P-specific IgG antibodies at 8 days post-microinjection. Actin was used as a loading control and detected with β-actin antibodies.

To further analyze the role of microtubules in the axonal transport of RYSV through the CNS of *N. cincticeps*, we first performed preliminary experiments to determine the optimum concentration of colchicine for microinjection into live *N. cincticeps* individuals. A total of 50 μg/ml colchicine did not significantly affect the growth of leafhopper (Supplementary Figure S4). We then microinjected third-instar *N. cincticeps* nymphs with either a RYSV inoculum containing 50 μg/ml colchicine or a RYSV inoculum diluted with the same volume of 0.01 M phosphate-buffered saline (PBS, pH 7.2) as the control treatment. At different days (3, 6, and 8 days) post-microinjection, we used immunofluorescence microscopy to reveal the effects of tubulin inhibitor on RYSV infection (Figure 6A). At 8 days post-microinjection, we observed that RYSV had proliferated in the abdominal ganglion, thoracic ganglion and brain of about 24% of the leafhoppers treated with colchicine, while RYSV proliferation was detected in 79% of those in the control group (Figure 6A and Table 2). Additionally, while the adjacent salivary glands were infected by RYSV in about 32% of leafhoppers microinjected with the RYSV solution, the virus had only invaded the salivary glands of about 13% of leafhoppers microinjected with the tubulin inhibitor (Figure 6A and Table 2). These results show that the microinjection of colchicine efficiently restricted viral infection of the CNS and adjacent salivary glands in most leafhoppers.

**Table 2:** Treatment with colchicine via microinjection suppressed the viral dissemination through the CNS and prevented viral infection of the salivary glands of *N. cincticeps.*

Treatment^a^	No. of whole CNSs containing the viral antigen	No. of salivary glands containing the viral antigen
	(*n* = 30)^b^	(*n* = 30)^c^
Control	23.67 ± 1.45	9.33 ± 0.33
Colchicine	10.33 ± 1.20	4 ± 0.58
*P*-value^d^	0.0021	0.0013

We next performed a reverse transcription quantitative PCR (RT-qPCR) assay to measure the effect of colchicine on RYSV infection at the transcript level at various times (1, 3, 5, 7, 9, and 11 days) post-microinjection. We found that at 1 day post-microinjection, the viral titers of the two samples were the same, making it clear that the abundance of both *N* and *M* gene transcripts was significantly reduced in the colchicine-treated group compared with the control group over time (Figure 6B). Moreover, the result of the immunoblot assay confirmed that the accumulation of viral proteins in the heads of viruliferous insects was also significantly reduced after the colchicine treatment at 8 days post-microinjection (Figure 6C). We also found that actin inhibitor could slightly suppress RYSV infection in its insect leafhoppers (Supplementary Figure S5). Based on the results above, it is clear that the microtubules facilitate the spread of RYSV virions within the CNS of the insect vector.

## Discussion

Persistently transmitted plant viruses must spread from their primary infection site in the insect alimentary canals to the salivary glands for transmission to the next host (Hogenhout et al., 2008; Bragard et al., 2013; Blanc et al., 2014; Wei and Li, 2016; Whitfield et al., 2018). It has been generally assumed that viruses pass from the midgut into the tissues or organs of the vector through the hemolymph. This common infection route could also present during RYSV infection in leafhopper, due to the identification of RYSV virion in both the filter chamber and hemolymph (data not shown). In our study, the neurotropic nature of RYSV in *N. cincticeps* suggests that an alternative non-hemolymph pathway could exist, allowing the rapid spread of viruses from the initial infection site in the midgut epithelium to the salivary glands via the nerve networks (Hirumi et al., 1967; Ammar and Hogenhout, 2008; Figure 1G,H). This would enable more efficient viral transmission by the insect vectors.

Here, we provide the first evidence, to our knowledge, that rapid axonal virus transport is used by neurotropic plant rhabdoviruses within their insect vectors. Axonal virus transport requires virions to attach to and enter the axons, travel along microtubules over long distances, and egress from the axons to infect other parts of the nervous system and eventually other organs. We have shown that the non-enveloped particles of RYSV can travel along the microtubules of the CNS axons in their leafhopper vector. Generally, the M protein of the rhabdoviruses form the outer capsid of non-enveloped virion particles and helps to condense the internal helical ribonucleoprotein complex comprising the RNA genome and the N protein, while the G protein serves to bind the enveloped virions to host cell receptors (Gaedigk et al., 1986; Jayakar et al., 2004; Jackson et al., 2005; Wang et al., 2015). We have further demonstrated that the M protein, but not the N or G proteins, of RYSV interacts specifically with tubulin, the main component of the neuronal microtubules in its insect vector. Treatment with drugs that inhibit the polymerization of the microtubule filaments abolished the ability of RYSV to spread in cultured insect vector cells and in the CNS of insect vectors, which simultaneously decreased the viral infection of their salivary glands. The function of actin in the axonal transport of RYSV was also evaluated in our study with the actin disrupting drug lantrunculin A. As expected, the disruption of actin did not significantly inhibit the infection of RYSV (Supplementary Figure S5).

Our results reveal a previously undescribed aspect of axonal transport, whereby a non-enveloped plant rhabdovirus may uses its M protein to move along the microtubule filaments in the axon of its insect host to facilitate long-distance viral spread. Of course, our current result could not rule out the role of G protein in axon transport of RYSV in leafhopper. Notably, a different mechanism of axonal virus transport was previously described for the neuroinvasion of the rabies rhabdovirus into its vertebrate animal hosts. In that system, enveloped rabies particles are enclosed in endosomes, then travel along the microtubule within the host axons, probably in a dynein-dependent manner (Tsiang, 1979; Klingen et al., 2008; Gluska et al., 2014). Our current model shows that the interaction of the M protein on the outer capsid of non-enveloped RYSV particles with the axon microtubules may confer a selective advantage, allowing the rapid transport of the smooth, bullet-shaped viruses. By contrast, the glycoprotein spikes on the surface of enveloped RYSV particles may confer a selective advantage for binding to the cell receptors on the axonal membrane and entering the axoplasm. Thus, the preferred axonal transport of rhabdoviruses may involve the “skating” of non-enveloped virions along the long axonal microtubules. There exists a certain portion of enveloped virions in the CNS axons, and it would be meaningful to explore whether a motor protein is incorporated into the axonal transport of RYSV-enveloped virion. The infection of RYSV was obviously inhibited when the microtubules were disrupted after the treatment of either Vincristine or Colchicine in VCMs which were derived from cultured leafhopper embryonic cells (Figure 5C). This result suggested that RYSV may also travel along microtubules of non-nerve cells by the interaction of M protein and tubulin protein.

RYSV is transmitted efficiently to plant hosts after its multiplication to a high viral titer in the CNS of its vector, in which its infection is apparent and persistent (Chiu, 1982). This persistent infection does not cause significant microtubule degeneration or cytopathologic changes in the virus-infected insect CNS, however, the neuroinvasive viruses of vertebrate animals generally cause microtubule degeneration, cellular pathogenesis, and even neurologic disease or similar neuroinflammatory disorders (McArthur et al., 2005; Spudich and González-Scarano, 2012; Avdoshina et al., 2016, 2017; Leibovitch and Jacobson, 2018). For example, the viral envelope glycoprotein of HIV binds to neuronal microtubules and alters the post-translational modifications of tubulin, leading to neuronal dysfunction (McArthur et al., 2005; Spudich and González-Scarano, 2012; Avdoshina et al., 2017). It is therefore useful to explore how RYSV infection avoids causing neurologic dysfunction in its insect vectors. In previous work, we have shown that a conserved small interfering RNA (siRNA) antiviral pathway in insects is activated to control excessive viral replication, ensuring insect vector survival and the persistent transmission of viruses (Lan et al., 2015, 2016). It is valuable to investigate whether antiviral siRNA pathways could restrict viral replication in the insect CNS to avoid neurologic dysfunction in RYSV-infected insect vectors. Additionally, the CNS is an important hormone-secretory system affecting a variety of physiological processes, including embryogenesis, post-embryonic development, behavior, water balance, polymorphism, mating, reproduction, and diapause (Penzlin, 1985). RYSV infection in the insect CNS may therefore influence the secretion of hormones such as dopamine and polyamines, which might manipulate the behavior of the insects to benefit viral persistent transmission, however, this hypothesis needs further validation. These findings therefore lay a foundation for the further investigation of the mechanisms underlying axonal virus transport and its ecological significance, as well as providing novel insights into the development of efficient approaches to attenuate viral epidemics by targeting axonal virus transport.

## Author Contributions

X-FZ and TW conceived and designed the experiments. HW, JW, QZ, TZ, and YZ performed the experiments. HW, JW, and XZ analyzed the data. HW, X-FZ, and TW wrote the manuscript. All authors read and approved the final manuscript.

## Conflict of Interest Statement

The authors declare that the research was conducted in the absence of any commercial or financial relationships that could be construed as a potential conflict of interest.
