# Supplementary material for: Rice Yellow Stunt Nucleorhabdovirus Matrix Protein Mediates Viral Axonal Transport in the Central Nervous System of Its Insect Vector
Source: Front Microbiol. 2019 May 9;10:939. doi: 10.3389/fmicb.2019.00939 (PMC6521124; doi:10.3389/fmicb.2019.00939)
Supplement: Supplementary file 1 [file Data_Sheet_1.PDF]

SUPPLEMENTARY MATERIALS

Rice yellow stunt nucleorhabdovirus matrix protein mediates viral axonal transport in the central nervous system of its insect vector

Haitao Wang [1,2], Juan Wang [1], Qian Zhang[1,2], Tianbao Zeng [1,2], Yuemin Zheng[1,2], Hongyan Chen [1,2], Xiao-Feng Zhang [1,2,] *, Taiyun Wei [1,2,] *

***Correspondence:**

Taiyun Wei: weitaiyun@fafu.edu.cn

Xiao-Feng Zhang: zhangxiaofeng911@163.com

Materials and Methods

RYSV detection in hemolymph of *N. cincticeps*

For the hemolymph detection, the Second-instar *N. cincticeps* nymphs fed on RYSV infected rice plant at different days padp were anesthetized on ice for 5 min and placed on one glass slide. The metapedes were excised from the leafhoppers. The hemolymph was collected from the wound by capillary pipette and then was transferred onto the poly-lysine treated glass slide. The hemolymph samples were then fixed in 4% PFA at room temperature for 30 min, permeabilized at room temperature in 0.2% Triton X-100 for 15min, then immunolabeled with RYSV-N antibody conjugated to rhodamine (N-R), RYSV-P antibody conjugated to FITC (P-F) and DAPI. The samples were examined under a confocal microscopy.

Transmission electron microscopy

For the immunoelectron microscopy, ultrathin sections of leafhopper CNS were incubated with protein-G-specific IgG from rabbit and immunogold-labeled using goat antibodies against rabbit IgG conjugated with 10-nm gold particles (Sigma-Aldrich), then doubled stained with 2% uranyl acetate and 3% lead citrate. The distribution of RYSV particles in axons of the CNS was analyzed using an electron microscope. Total 45 ultrathin sections from 15 viruliferous *N. cincticeps* individuals (3 sections for each leafhopper) were counted. The electron observation assay was repeated three times. The presence of RYSV particles distributed in the entry or exit site of CNS axons was analyzed from10 viruliferous *N. cincticeps* individuals using an electron microscope. The samples were observed under an electron microscope (Hitachi H-7650).

Effects of microtubule inhibitor on the survival rate and eclosion rate of *N. cincticeps*

Microtubule inhibitor reagent colchicine (50 μg/ml) was microinjected into the third instar *N. cincticeps*. Another group of *N. cincticeps* was injected with 0.01 M PBS as a control. Both of the microinjected insects were reared on healthy rice seedlings separately and the survival rate or eclosion rate was counted at different days post microinjection.

Effects of actin inhibitor on RYSV infection in *N. cincticeps*

Third-instar nymphs were microinjected with actin inhibitor latruncunlin A (Final

concentration; 5 μM) mixing with RYSV inoculum. Another group of nymphs were microinjected with the RYSV solution diluted with the same volume of DMSO, as a control. The microinjected insects were reared on healthy rice seedlings. To trace the infection of RYSV in the CNS, 30 leafhoppers under each treatment were dissected at 8 days post microinjection, fixed in 4% PFA at room temperature for at least 8 h, and permeabilized at room temperature in 4% Triton X-100 in 0.01 M PBS buffer for 24 h, then immunolabeled with phalloidine-FITC (phalloidine-F) or virus-antibody-rhodamine (V-R), then examined with a confocal micaroscope.

Fig. S1

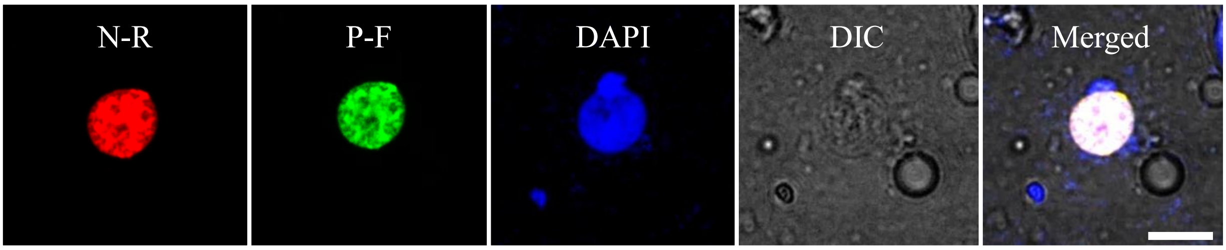

Figure S1. RYSV infected the hemolymph of *N. cincticeps*. The hemolymph of *N. cincticeps* were detected at different days padp, and the fluorenscence signals only detected at 8 padp. The hemolymph were immuno-labeled with RYSV N antibody conjugated to rhodamine (N-R, red), RYSV P antibody conjugated to FITC (P-F, green) and DAPI recognized the nuclei. Scale bar, 10 μm.

Fig. S2

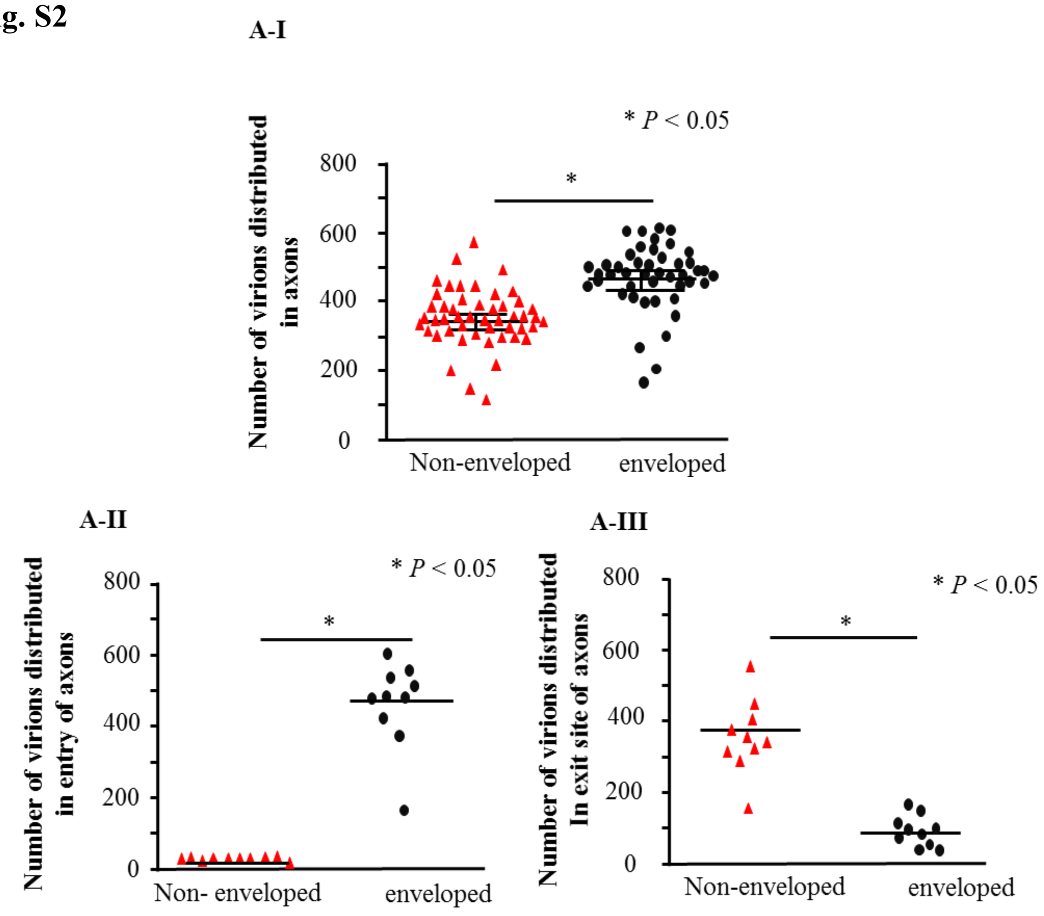

Figure S2. RYSV particles distributed in the axons of viral infected *N. cincticeps* CNS. (A-I) Number of enveloped and non-enveloped RYSV virions in the axons of the *N. cincticeps* CNS. Virions were counted in the axons observed in three ultrathin CNS sections taken from each 15 viruliferous *N. cincticeps* individual using an electron microscope, three replicates were performed. The means of virions numbers (non-enveloped: 361.5; enveloped: 565.8). P values were estimated using a Tukey's honest significant difference (HSD) test. (A-II), Number of viral particles in the entry site of CNS axons. The means of virions numbers (non-enveloped: 5; enveloped: 494). (A-III), Number of viral particles in the exit site of *N. cincticeps* CNS axons. The means of virions numbers (non-enveloped: 377; enveloped: 104). Each dot or triangle represents one insect.

Fig. S3

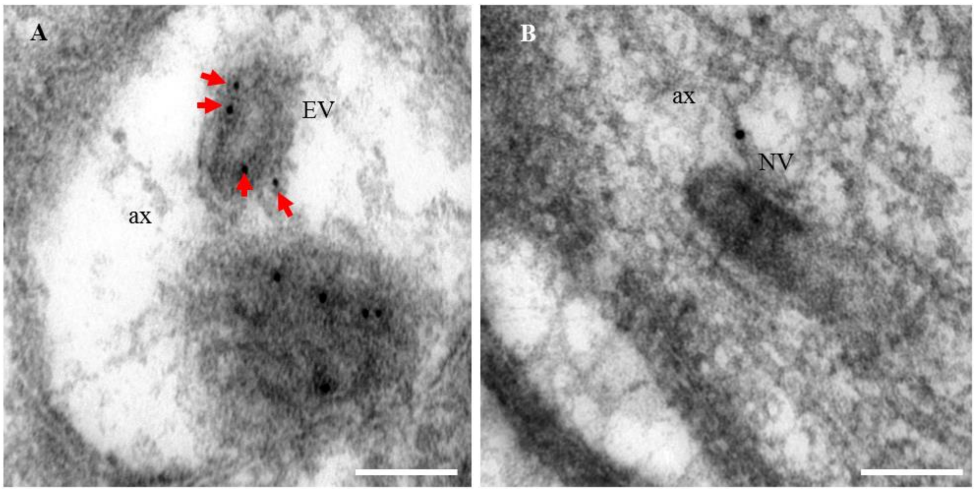

Figure S3. RYSV infected axons of *N. cincticeps* CNS immunolabeled with RYSV G antibody. (A) The enveloped viral particle reacted specifically with G antibody; (B) The non-enveloped viral particle could not be labeled with G antibody. EV, enveloped viral particle; NV, non-enveloped viral particle. Scale bars, 100 nm.

Fig. S4

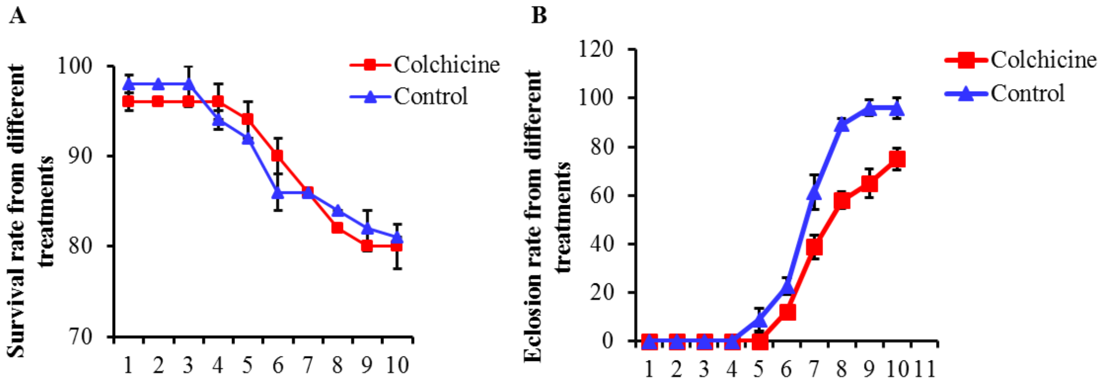

Figure S4. The effect of microtubule inhibitor (colchicine) on the life activity of *N. cincticeps*. The survival rates (A) and the eclosion rate (B) of *N. cincticeps* at different days post reagents microinjection.

Fig. S5

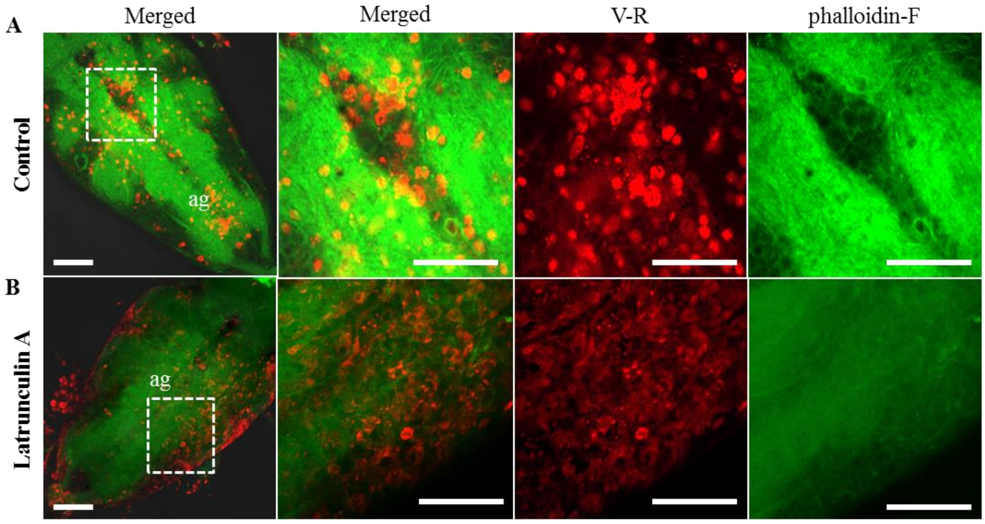

Figure S5. The effect of actin inhibitor (Latrunculin A) on RYSV infection in *N. cincticeps*. The CNS tissues from two treatment (Control, A; latrunculin A, B) were dissected, fixed and immuno-labeled with RYSV antibody conjugated to rhodamine (V-R, red) and phalloidin-FITC (green). Scale bars, 50 µm.
